# Practical Security Analysis of Reference Pulses for Continuous-Variable Quantum Key Distribution

**DOI:** 10.1038/s41598-019-54249-0

**Published:** 2019-12-03

**Authors:** Wei Zhao, Ronghua Shi, Duan Huang

**Affiliations:** School of Computer Science and Engineering, Central South University, Changsha, 410083 China

**Keywords:** Physics, Quantum physics

## Abstract

By manipulating the reference pulses amplitude, a security vulnerability is caused by self-reference continuous-variable quantum key distribution. In this paper, we formalize an attack strategy for reference pulses, showing that the proposed attack can compromise the practical security of CVQKD protocol. In this scheme, before the beam splitter attack, Eve intercepts the reference pulses emitted by Alice, using Bayesian algorithm to estimate phase shifts. Subsequently, other reference pulses are re-prepared and resubmitted to Bob. In simulations, Bayesian algorithm effectively estimates the phase drifts and has the high robustness to noise. Therefore, the eavesdropper can bias the excess noise due to the intercept-resend attack and the beam splitter attack. And Alice and Bob believe that their excess noise is below the null key threshold and can still share a secret key. Consequently, the proposed attack shows that its practical security can be compromised by transmitting the reference pulses in the continuous-variable quantum key distribution protocol.

## Introduction

Quantum key distribution (QKD) is one of the advanced technology to date, and the security of the shared keys is guaranteed by the quantum mechanics^1–5^. Allowing two authenticated parties to establish secret keys in an insecure channel, QKD provides a secure way. In the discrete-variable quantum key distribution (DVQKD), several significant achievements have been achieved^6–12^. For implementing DVQKD, Pan *et al*. has launched a low-Earth-orbit satellite, with key rate around 20 orders of magnitudes greater than optical fiber^13^. For decades, continuous-variable quantum key distribution (CVQKD) without the requirement of single-photon detection, has made significant progress of QKD research^14–16^. However, there is a fundamental limit on the maximum number of secret bits that can be generated by two remote parties. This limit is the secret key capacity of the lossy channel, which also known as the PLOB bound^17^. In this general context, Gaussian-modulated coherent state (GMCS) protocol has been experimentally achieved both in long distance and high speed^18–21^. Moreover, the protocol has already proved secure in the asymptotic regime^22,23^ and finite-size regime^24,25^. To be specific, the signal pulses and local oscillator (LO) pulses are simultaneously prepared by Alice in the GMCS protocol. In other words, the LO is deemed as a fixed phase reference for the signal detection, which can reduce the phase noise.

Nevertheless, the transmission of the LO brings about several limitations^26–29^. Firstly, the LO may be controlled and modified by eavesdropper. Eve may launch attacks by manipulating the LO, such as LO fluctuation attacks^30^, calibration attacks^31^, saturation attacks^32^ and wavelength attacks^28,33^. Secondly, the complicated techniques of multiplexing and de-multiplexing are necessary to transmit and separate the two pulses, respectively. Thirdly, sending the strong LO pulses can reduce the transmission efficiency. In order to solve these problems, self-reference CVQKD protocols are proposed, which can generate the LO locally at Bob’s side. Besides, several protocols have been proposed to solve the flaws of phase drift in the self-reference CVQKD protocol^34–36^.

Recently, Qin *et al*. formalize an attack strategy; Eve cuts down the quantum channel and inserts an external light into the self-reference CVQKD system^37^. However, the countermeasure for this attack is proposed in the reference^38^. Shengjun *et al*. propose a reference pulse attack, which can exploit the phase estimation error associated with the reference pulses to attack the self-reference CVQKD protocol^39^. Besides, Pereira *et al*. consider an attack on a coherent-state protocol; Eve not only taps the main communication channel but also hacks AliceâĂŹs device^40^. Nevertheless, there are some defects in theoretical analysis and it is hard to achieve. Inspired by side channel attack, we formalize the attack strategy for discrete-modulated and Gaussian-modulated self-reference CVQKD, respectively. The attack works as follows: Eve increases her amount of beam splitter attack on the quantum signal, which inevitably increasing the excess noise. Before the beam splitter attack, she utilizes the intercept-resend attack and the Bayesian algorithm to decrease the phase estimation error noise. Therefore, Eve can bias the excess noise due to the beam splitter attack and the intercept-resend attack, and Alice and Bob believe their excess noise estimation is below the null key threshold and they can still share a secret key. To intercept-resend attack, that is, Eve can monitor and intercept the reference pulses emitted by Alice. Subsequently, she measures each reference pulse and then estimates the phase drift using the Bayesian algorithm. After phase estimation and compensation operation, she re-prepares and resubmits another reference pulses, which is sent to Bob. What’s more, we propose to utilize Bayesian algorithm to estimate the reference pulses. The algorithm not only can obtain a confidence interval, but also has robustness to noise. Therefore, the algorithm can increase the accuracy of phase estimation for Eve. This series of operations can cause a security vulnerabilities through the manipulation of the reference pulses.

## Practical Security Analysis

**Figure 1:**
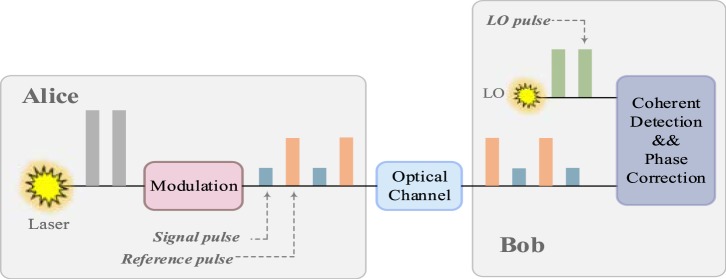
The self-reference CVQKD protocol. Alice sequentially sends reference pulses and signal pulses. At the receiver, Bob uses its own LO pulses to perform coherent detection. Here, LO represents the local oscillator.

In this section, we start by describing the protocol of the self-reference CVQKD. Subsequently, we analyze how Eve can intercept the reference pulses and estimate the phase drift for the discrete-modulated and Gaussian-modulated self-reference CVQKD, respectively. The self-reference CVQKD protocol, as shown in Fig. 1, consists of two parts, one is the coherent states preparation and propagation, and the other part is the coherent states detection and processing. At the transmitter, Alice prepares the Gaussian-modulated coherent states (or the discrete-modulated states) and then transmits to Bob. The arbitrary phase drift of the states will be inevitably induced through the quantum channel. Therefore, the phase reference pulses are necessary to transmit along with the signal pulses. As depicted in Fig. 1, the reference pulses and signal pulses are sent by Alice alternatively and periodically. At the receiver, Bob can utilize the relatively strong reference pulses to estimate the phase^35^. In theory, quantum phase noise $\Delta \varphi$ between the two users can be written as^41^

1 $$\Delta \varphi \approx \frac{2\pi }{s}\Delta vL,$$

where $L$ represents the length of the fiber, $\Delta v$ is the difference frequency between the user’s lasers, and $s$ denotes the speed of light in the fiber. Although several protocols are proposed to the phase compensation, the strong reference pulses are still indispensable in the self-reference CVQKD. However, propagating the relatively strong reference pulses may result in security vulnerabilities.

**Figure 2:**
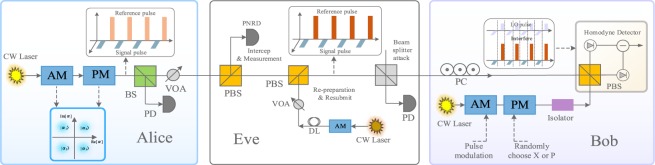
The practical security analysis of reference pulses for the CVQKD protocol with discrete modulation. CW laser, continuous wave laser; BS, beam splitter; PD, photodetector; VOA, variable optical attenuator; PBS, polarizing beam splitter; Att., attenuator; PNRD, photon number resolving detector; DL, delay line; PC, polarization controller; AM-PM, amplitude and phase modulation.

In what follows, we analyze the practical security of reference pulses for CVQKD with discrete modulation. In this scenario, Eve has the ability to monitor and intercept the reference pulses from Alice to Bob. Basically, Eve measures each reference pulse and estimates the phase drift emitted by Alice. After the phase compensation, she re-prepares and resubmits reference pulses, which are sent to Bob. Next, we introduce how Eve employs Bayesian algorithm to estimate the phase drift of the intercepted reference pulses. Without loss of generality, we analyze the four-state self-reference CVQKD protocol^42–44^. The four-state can be denoted as $\left|\,{\alpha }_{k}\right\rangle =\left|\,\alpha {e}^{i(2k+1)\pi /4}\right\rangle$ with $k\in \{0,1,2,3\}$, and the modulation variance is $V=1+2{\alpha }^{2}$. When Eve intercepts reference pulses through the noisy channel, as shown in Fig. 2, the photon number resolving detector (PNRD) performs the measurement operation. Specifically, the measurement outcomes are denoted as ${\mathcal E} \in \{\left|\,{\alpha }_{0}\right\rangle ,\left|\,{\alpha }_{1}\right\rangle ,\left|\,{\alpha }_{2}\right\rangle ,\left|\,{\alpha }_{3}\right\rangle \}$, and the total number of detected photons are described as ${\mathscr E}={\sum }_{i=0}^{3}{n}_{i}$, where ${n}_{i}$ symbolizes the detected photon of state $\left|\,{\alpha }_{i}\right\rangle$. Then, we provide the critical process of Bayesian estimation. Above all, in order to estimate the correct eigenphase, an initial prior probability distribution ${\mathscr P}(\varphi )$ is provided to express the confidence interval that the current hypotheses is the correct eigenphase. Subsequently, the mean $\mu$ and its standard derivation $\sigma$ are updated on the basis of the measurement results of PNRD. What’s more, the posterior probability distribution can be calculated as^45,46^

2 $${\mathscr P}(\varphi |  {\mathcal E} )=\frac{{\mathscr P}( {\mathcal E} | \varphi ){\mathscr P}(\varphi )}{\int {\mathscr P}( {\mathcal E} | \varphi ){\mathscr P}(\varphi ){\rm{d}}\varphi }.$$

Specifically, some particles drawn from the ${\mathscr P}(\varphi )$ mismatch the likelihood function, which will be discarded later. The likelihood function is defined as ${\mathscr P}(\left|\,{\alpha }_{0}\right\rangle | \varphi )=\frac{1}{4}(1+{e}^{-{\Delta }^{2}}\cos (\varphi ))$, ${\mathscr P}(\left|\,{\alpha }_{1}\right\rangle | \varphi )=\frac{1}{4}(1+{e}^{-{\Delta }^{2}}\sin (\varphi ))$, ${\mathscr P}(\left|\,{\alpha }_{2}\right\rangle | \varphi )=\frac{1}{4}(1-{e}^{-{\Delta }^{2}}\cos (\varphi ))$ and ${\mathscr P}(\left|\,{\alpha }_{3}\right\rangle | \varphi )=\frac{1}{4}(1-{e}^{-{\Delta }^{2}}\sin (\varphi ))$. Without loss of generality, the qubit undergoes a phase diffusion process whose amplitude is characterized by the parameter $\Delta$. After obtaining the posterior distribution in Eq. 2, we set the posterior probability distribution to equal the prior probability distribution. This updating program can be deemed as the iterative processing for each of the emulation.

**Figure 3:**
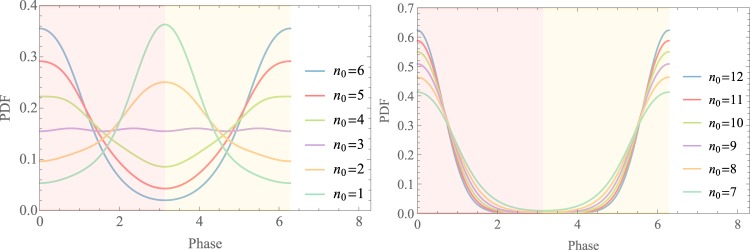
Probability density function (PDF) versus the phase drift.

**Figure 4:**
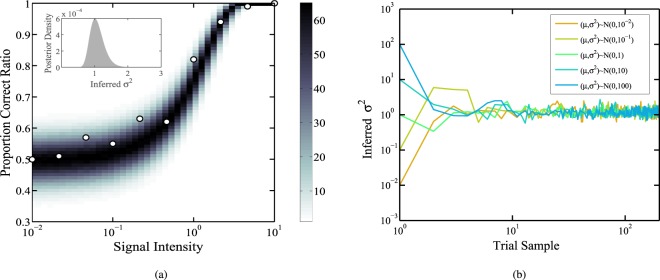
(**a**) The prediction model. A set of data points are performed in the simulation to estimate the phase variance by Bayesian phase estimation algorithm. (**b**) The parameter procedure with the different ${\sigma }^{2}$.

Subsequently, we provide the mathematical definition of probability density function (PDF) to illustrate the relation between the phase shift and the detected photons ${\mathscr E}$. The PDF is defined as ${{\mathscr P}}_{B}(\varphi ;{\mathscr E})=\frac{{\sum }_{i=0}^{3}{\mathscr P}{(\left|{\alpha }_{i}\right\rangle | \varphi )}^{{n}_{i}}}{ {\mathcal M} }$, where ${\mathcal M}$ is the normalization factor satisfies with ${\int }_{0}^{2\pi }{{\mathscr P}}_{B}(\varphi ;{\mathscr E}){\rm{d}}\varphi =1$. The simulation results of phase drift are depicted in Fig. 3. The two subgraphs illustrate that, the phase drift tends to zero if we increase the number of ${n}_{0}$ (where ${n}_{1}$, ${n}_{2}$ and ${n}_{3}$ are constant). Here, ${n}_{i}$ is defined in the previous paragraph. Consequently, increasing the detected photons for intercepted reference pulses can improve the accuracy of phase estimation, thus reducing the possibility of phase shift. Next, we describe the main implementation steps of the Bayesian algorithm. In the initial interation of the algorithm, a prior distribution ${\mathscr E}({\mu }_{0},{\sigma }_{0})$ is to express the confidence interval. Then, the dataset is utilized to update the $\mu$ and $\sigma$ of the posterior probability distribution in accordance with Bayes’ theorem. The parameter estimation for the inferred ${\sigma }^{2}$ of the posterior probability density is simulated in Fig. 4a, and the shaded region stands for the proportion correct ratio of the predicted trials. In other words, increasing the signal intensity level can improve the proportion correct of concentration. As shown in Fig. 4b, we utilize the different initial ${\sigma }^{2}$ to simulate and test that the performance is insensitive to the initial ${\sigma }^{2}$. In other words, the Bayesian algorithm has the high robustness. Furthermore, the main steps of the algorithm^45,47,48^ are described in the Appendix.

**Figure 5:**
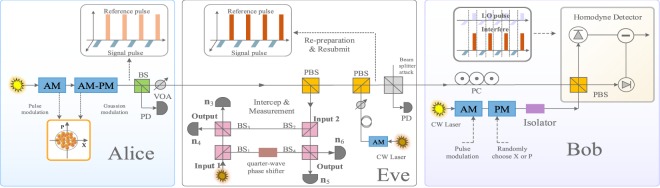
The practical security analysis of reference pulses for the CVQKD protocol with Gaussian modulation. CW laser, continuous wave laser; BS, beam splitter; PD, photodetector; VOA, variable optical attenuator; PBS, polarizing beam splitter; Att., attenuator; PNRD, photon number resolving detector; DL, delay line; PC, polarization controller; AM, amplitude modulation; PM, phase modulation.

By comparison to the discrete-modulated CVQKD protocol, the Gaussian-modulated CVQKD protocol is more complicated. We adopt the Mach-Zehnder (MZ) interferometer to estimate the phase drifts^49–53^. The Mach-Zehnder interferometer, as shown in Fig. 5, has two inputs labelled $1$ and $2$, one input is the intercepted reference pulses, and the other is the coherent light source. The two inputs are combined in two beamsplitters ($B{S}_{1}$ and $B{S}_{2}$) and two internal arms. On the one of the branches for output, a beamsplitter ($B{S}_{3}$) has two outputs labelled as $3$ and $4$. On the other branch, a beamsplitter ($B{S}_{3}$) has two outputs labelled as $5$ and $6$. According to the mentioned above, photodetectors are applied to outputs and respond to intensities ${I}_{k}$. Therefore, we integrate over some observations $T$, and define the parameter ${W}_{k}={\int }_{T}{I}_{k}\,{\rm{d}}t$ with $k\in \left\{3,4,5,6\right\}$. Particularly, the parameter ${W}_{k}$ can be substituted by the integer ${n}_{k}$, where ${n}_{k}$ represents the photodetection result in the time interval $T$. Based on the NFM theory (Noh, Fougères and Mandel)^51^, the unambiguous value of phase $\varphi$ is estimated as

3 $$\cos \varphi =\frac{{n}_{3}-{n}_{4}}{{\left[{({n}_{3}-{n}_{4})}^{2}+{({n}_{5}-{n}_{6})}^{2}\right]}^{1/2}}\,,\,\sin \varphi =\frac{{n}_{5}-{n}_{6}}{{\left[{({n}_{3}-{n}_{4})}^{2}+{({n}_{5}-{n}_{6})}^{2}\right]}^{1/2}}.$$

When the photon number ${\bar{n}}_{{\rm{in}}}$ at input $1$ and $2$ is determined, the outputs $3\, \sim \,6$ have the mean values ${\bar{n}}_{3}={\bar{n}}_{{\rm{in}}}(1+V\cos \varphi )$, ${\bar{n}}_{4}={\bar{n}}_{{\rm{in}}}(1-V\cos \varphi )$, ${\bar{n}}_{5}={\bar{n}}_{{\rm{in}}}(1+V\sin \varphi )$ and ${\bar{n}}_{6}={\bar{n}}_{{\rm{in}}}(1-V\sin \varphi )$, where the maximum likelihood estimate can be given by

4 $$\widehat{V}=\sqrt{(\frac{{n}_{3}-{n}_{4}}{{n}_{3}+{n}_{4}})+{(\frac{{n}_{5}-{n}_{6}}{{n}_{5}+{n}_{6}})}^{2}}.$$

Consequently, based on the Poissonian distribution of photoncount and mean value ${\bar{n}}_{{\rm{in}}}$, the likelihood of $\varphi$ and ${\bar{n}}_{{\rm{in}}}$ is

5 $${\mathscr P}({\bf{n}}| \varphi ,{\bar{n}}_{{\rm{in}}})\propto {\left({\bar{n}}_{{\rm{in}}}\right)}^{\sum {n}_{k}}{{\rm{e}}}^{-2{\bar{n}}_{{\rm{in}}}}{(1+V\cos \varphi )}^{{n}_{3}}{(1-V\cos \varphi )}^{{n}_{4}}{(1+V\sin \varphi )}^{{n}_{5}}{(1-V\sin \varphi )}^{{n}_{6}},$$

with the notation ${\bf{n}}=\left[{n}_{3},{n}_{4},{n}_{5},{n}_{6}\right]$. Assuming that ${\bar{n}}_{{\rm{in}}}$ is independent of $\varphi$ and $V$, we have

6 $${\mathscr P}({\bf{n}}| \varphi )=\int {\mathscr P}({\bf{n}}| \varphi ,{\bar{n}}_{{\rm{in}}}){\mathscr P}({\bar{n}}_{{\rm{in}}}){\rm{d}}{\bar{n}}_{{\rm{in}}}\propto {(1+V\cos \varphi )}^{{n}_{3}}{(1-V\cos \varphi )}^{{n}_{4}}{(1+V\sin \varphi )}^{{n}_{5}}{(1-V\sin \varphi )}^{{n}_{6}}.$$

Consequently, the posterior probability distribution takes the following form

7 $${\mathscr P}(\varphi | {\bf{n}})=\frac{{\mathscr P}({\bf{n}}| \varphi ){\mathscr P}(\varphi )}{\int {\mathscr P}({\bf{n}}| \varphi ){\mathscr P}(\varphi ){\rm{d}}\varphi },$$

which is in accordance with the Eq. 2 of four-state self-reference CVQKD protocol.

## Performance Analysis

First of all, we define transmission probability and transition probability to analyze the performance of the reference pulses for the discrete modulation protocol. In the four-state self-reference CVQKD protocol, there are four kinds of the intercepted encoding phase, namely the ${\varphi }_{i}=\frac{(2k+1)\pi }{4}$, with $k\in \left\{0,1,2,3\right\}$. According to the likelihood function, if the transmission states are in consistent with the measurement outcome ${\mathcal E}$, the equation can be simplified as

8 $$\begin{array}{rcl}{\mathscr P}(\left|\,{\alpha }_{0}\right\rangle | {\varphi }_{0}) & = & \frac{1}{4}(1+{e}^{-{\Delta }^{2}}\cos (\pi /4))\\  & = & \frac{1}{4}(1+\frac{\sqrt{2}}{2}{e}^{-{\Delta }^{2}}),\\ {\mathscr P}(\left|\,{\alpha }_{1}\right\rangle | {\varphi }_{1}) & = & \frac{1}{4}(1+{e}^{-{\Delta }^{2}}\sin (3\pi /4))\\  & = & \frac{1}{4}(1+\frac{\sqrt{2}}{2}{e}^{-{\Delta }^{2}}),\\ {\mathscr P}(\left|\,{\alpha }_{2}\right\rangle | {\varphi }_{2}) & = & \frac{1}{4}(1-{e}^{-{\Delta }^{2}}\cos (5\pi /4))\\  & = & \frac{1}{4}(1+\frac{\sqrt{2}}{2}{e}^{-{\Delta }^{2}}),\\ {\mathscr P}(\left|\,{\alpha }_{3}\right\rangle | {\varphi }_{3}) & = & \frac{1}{4}(1-{e}^{-{\Delta }^{2}}\sin (7\pi /4))\\  & = & \frac{1}{4}(1+\frac{\sqrt{2}}{2}{e}^{-{\Delta }^{2}}).\end{array}$$

**Figure 6:**
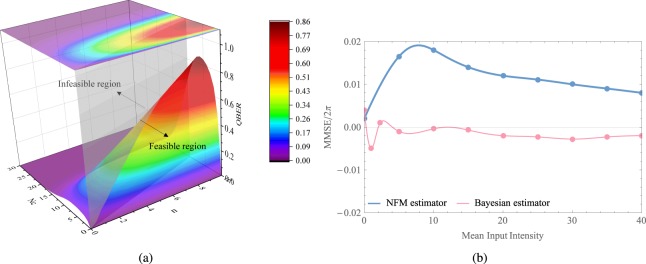
(**a**) The QBER of the intercept-resend attack with the discrete modulation protocol. (**b**) Difference in minimum mean squared error for the two phase estimation schemes, namely the NFM estimator protocol^51,53,54^, and Bayesian estimator protocol, respectively. Here, the phase shift is set as $\pi /3$.

Therefore, the transmission probability can be defined as ${{\mathscr P}}_{ii}={\mathscr P}(\left|\,{\alpha }_{i}\right\rangle | {\varphi }_{i})$ with $i\in \left\{0,1,2,3\right\}$, which satisfies the constraint ${\sum }_{j=0}^{3}{\mathscr P}(\left|\,{\alpha }_{i}\right\rangle | {\varphi }_{j})=1$. Moreover, the transition probability can be expressed as ${{\mathscr P}}_{ij}=1-{{\mathscr P}}_{ii}$ with $i\ne j$. Subsequently, the QBER for Eve can be calculated as $QBER={\sum }_{n}{{\mathscr Q}}_{n}{ {\mathcal R} }_{n}$, with the notation

9 $${{\mathscr Q}}_{n}=\left\{\begin{array}{l}{\sum }_{k=(n+1)/2}^{n}{{\rm{C}}}_{n}^{k}{{\mathscr P}}_{ij}^{k}{(1-{{\mathscr P}}_{ij})}^{n-k}n\,{\rm{is}}\,{\rm{odd}},\\ {\sum }_{k=(n+2)/2}^{n}{{\rm{C}}}_{n}^{k}{{\mathscr P}}_{ij}^{k}{(1-{{\mathscr P}}_{ij})}^{n-k}+\frac{1}{2}{{\rm{C}}}_{n}^{n/2}{{\mathscr P}}_{ij}^{n/2}{(1-{{\mathscr P}}_{ij})}^{n/2}\,\,\,\,\,n\,{\rm{is}}\,{\rm{even}},\end{array}\right.$$

and ${ {\mathcal R} }_{n}=\frac{{e}^{-{N}_{c}}\cdot {N}_{c}^{n}}{n!},{C}_{n}^{k}=\frac{n!}{k!(n-k)!},n!=1\times 2\times 3\times \cdots (n-1)\times n$. Here, $n$ and ${N}_{c}$ symbolize the transmitted photons (at the Alice’s side) and detected photons (at the Eve’s side) per pulse, respectively. Figure 6a depicts the QBER for Eve with the discrete modulation protocol. Considering the existence of noise in the channel, two parameters are restricted with $n > {N}_{c}$. According to the result, we can see that, increasing the number of $n$ and ${N}_{c}$ will improve the QBER of the intercept-resend attack for Eve.

In the following, we analyze the performance of the Bayesian estimator for the Gaussian-modulated self-reference CVQKD protocol. The Bayesian cost can be defined by the relation

10 $$\bar{ {\mathcal B} }({\bf{n}})={\int }_{{\varphi }^{^{\prime} }-\pi }^{{\varphi }^{^{\prime} }+\pi }{({\varphi }^{^{\prime} }-\varphi )}^{2}{\mathscr P}(\varphi | {\bf{n}})\,{\rm{d}}\varphi .$$

Particularly, if a suitable initial phase value ${\varphi }^{^{\prime} }$ is given, the minimum mean squared error (MMSE) estimator of ${\varphi }^{^{\prime} }$ has the form

11 $${\rm{MMSE}}={\int }_{{\varphi }^{^{\prime} }-\pi }^{{\varphi }^{^{\prime} }+\pi }\varphi {\mathscr P}(\varphi | {\bf{n}})\,{\rm{d}}\varphi ,$$

where ${\varphi }^{^{\prime} }$ can be initialized with the maximum likelihood (ML) estimate, and it can be defined as

12 $${{\rm{e}}}^{i{\varphi }^{^{\prime} }}=\frac{1}{\widehat{V}}\left[\frac{{n}_{3}-{n}_{4}}{{n}_{3}+{n}_{4}}+i\frac{{n}_{5}-{n}_{6}}{{n}_{5}+{n}_{6}}\right].$$

Figure 6b simulates the performance of the different phase estimation schemes, in particular, the input intensity ${\bar{n}}_{{\rm{in}}}$ is simulated for the fixed phase shift $\frac{\pi }{3}$. Specifically, the blue line denotes the NFM estimator^51,53,54^. Consequently, the Bayesian estimator outperforms NFM estimator.

Although the intercept-resend attack can compromise the practical security of QKD, the two remote participants can discover eavesdropping by the following method. At the receiver, Bob randomly chooses the same number of quantum pulses and reference pulses as training signals^55^. By utilizing the training signals, we can estimate the phase compensation error on reference signals and quantum signals, respectively. If the phase compensation error on signal pulses is different from that on reference pulses, we can conclude that Eve’s attack is attached in the quantum channel.

## Conclusion

In this paper, we analyze a security vulnerability of strong reference pulses in the realistic self-reference CVQKD system. In this scenario, before the beam splitter attack, Eve intercepts the reference pulses emitted by Alice, and utilizing the Bayesian algorithm to estimate phase drifts of reference pulses. After phase estimation and compensation, she resubmits another reference pulses to Bob. The algorithm not only can obtain a well-motivated confidence interval, but also has robustness to noise. Thus, due to the intercept-resend attack and the beam splitter attack, Eve can bias the excess noise. Consequently, it shows that the practical security of the proposed attack can be compromised by transmitting the reference pulses in the continuous-variable quantum key distribution protocol.

## Appendix

In the following, we take the four-state self-reference CVQKD protocol as an example, to derive the expression of the secret key rate under the intercept-resend attack. Assuming that the phase noise of quantum channel is zero-mean with variance ${V}_{{\rm{ch}}}$, while the phase noise reduced by Eve’s Bayesian algorithm is zero-mean with variance ${V}_{{\rm{Bayes}}}$, the deviation of the actual phase compensation error can be given by ${V}_{s}={V}_{{\rm{ch}}}-{V}_{{\rm{Bayes}}}$. According to the imperfect phase compensation, the actual transmittance can be defined as ${T}_{\kappa }=\kappa T$, where $\kappa$ represents the phase estimation accuracy with $\kappa ={\left(1-\frac{1}{2}{V}_{s}\right)}^{2}$, and $T$ is the transmission efficiency. Besides, the actual excess noise can be expressed as ${\epsilon }_{\kappa }=\left[\epsilon +(1-\kappa )(V-1)\right]/\kappa$. Therefore, the total noise referred to the channel input can be expressed as ${\chi }_{{\rm{tot}}}^{\kappa }={\chi }_{{\rm{line}}}^{\kappa }+{\chi }_{\hom }/{T}_{\kappa }$, with the notation ${\chi }_{{\rm{line}}}=1/{T}_{\kappa }+{\epsilon }_{\kappa }-1$.

When Alice and Bob use reverse reconciliation, the secret key rate can be defined as

13 $$S=\beta I(A:B)-\chi (B:E),$$

where $\beta$ is the reconciliation efficiency, $I(A:B)$ is the mutual information between Alice and Bob, and $\chi (B:E)$ is the mutual information between Bob and Eve. Specifically, the mutual information $I(A:B)$ is given by^55^

14 $$I(A:B)=\frac{1}{2}{{\rm{\log }}}_{2}\frac{V+{\chi }_{{\rm{tot}}}^{\kappa }}{1+{\chi }_{{\rm{tot}}}^{\kappa }}.$$

The Holevo bound of the information between Eve and Bob is given by

15 $$\chi (B:E)=\mathop{\sum }\limits_{i=1}^{2}G\left(\frac{{\lambda }_{i}-1}{2}\right)-\mathop{\sum }\limits_{i=3}^{5}G\left(\frac{{\lambda }_{i}-1}{2}\right),$$

where $G(x)=(x+1){{\rm{\log }}}_{2}(x+1)-x{{\rm{\log }}}_{2}x$. The symplectic eigenvalues ${\lambda }_{1,2}$ are given by

16 $${\lambda }_{1,2}=\sqrt{\frac{1}{2}(A\pm \sqrt{{A}^{2}-4B})},$$

where

17 $$\begin{array}{ll}A & ={V}^{2}+{T}_{\kappa }^{2}(V+{{\chi }_{{\rm{line}}}^{\kappa }}^{2}-2{T}_{\kappa }{Z}_{4}^{2}),\\ B & ={({T}_{\kappa }{V}^{2}+{T}_{\kappa }V{\chi }_{{\rm{line}}}^{\kappa }-{T}_{\kappa }{Z}_{4}^{2})}^{2}.\end{array}$$

The symplectic eigenvalues ${\lambda }_{3,4}$ are given by

18 $${\lambda }_{3,4}=\sqrt{\frac{1}{2}(C\pm \sqrt{{C}^{2}-4D})},\,{\lambda }_{5}=1,$$

with the notation

19 $$\begin{array}{ll}C & =\frac{A{\chi }_{\hom }+V\sqrt{B}+{T}_{\kappa }(V+{\chi }_{{\rm{line}}}^{\kappa })}{{T}_{\kappa }(V+{\chi }_{{\rm{tot}}}^{\kappa })},\\ D & =\sqrt{B}\frac{V+\sqrt{B}{\chi }_{\hom }}{{T}_{\kappa }(V+{\chi }_{{\rm{tot}}}^{\kappa })}.\end{array}$$

**Figure 7:**
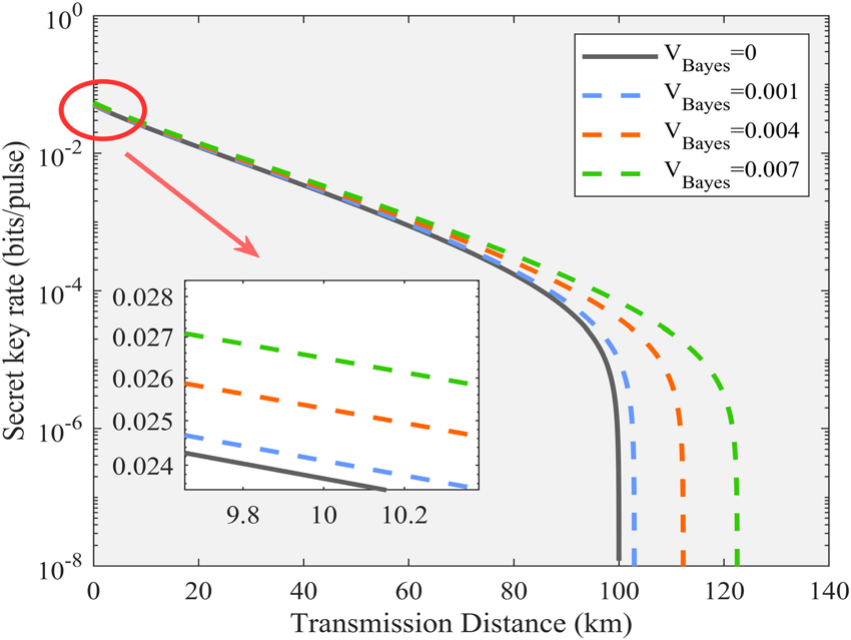
The theoretical and practical secret key rate of the four-state self-reference CVQKD under the intercept-resend attack. The phase noise reduced by Eve’s Bayesian algorithm submits to the normal distribution with variance $0.001$, $0.004$ and $0.007$ (${{\rm{rad}}}^{2}$) respectively. In particular, ${V}_{{\rm{Bayes}}}=0$ represents the original CVQKD protocol without the intercept-resend. Other parameters are summarized below: $\alpha =0.35$, ${v}_{{\rm{el}}}=0.001$, $\epsilon =0.01$, $\eta =0.6$, $\beta =0.95$ and ${V}_{{\rm{ch}}}=0.01({{\rm{rad}}}^{2})$.

Subsequently, we conduct numerical simulation using the realistic parameters. The parameters are summarized below: $\alpha =0.35$, ${v}_{{\rm{el}}}=0.001$, $\epsilon =0.01$, $\eta =0.6$, $\beta =0.95$ and ${V}_{{\rm{ch}}}=0.01({{\rm{rad}}}^{2})$. Here, the phase noise reduced by Eve’s Bayesian algorithm submits to the normal distribution with variance $0.001$, $0.004$ and $0.007$ (${{\rm{rad}}}^{2}$) respectively. In particular, ${V}_{{\rm{Bayes}}}=0$ represents the original CVQKD protocol without the intercept-resend. Fig. 7 is the simulation result in the asymptotic scenario. According to the simulation result, we can conclude that, the estimated key rate based on the intercept-resend and Bayesian algorithm is higher than the true security key rate. Therefore, the attack is effective in the self-reference CVQKD protocol. In our manuscript, the proposed algorithm is described as follows.

**Algorithm 1:**
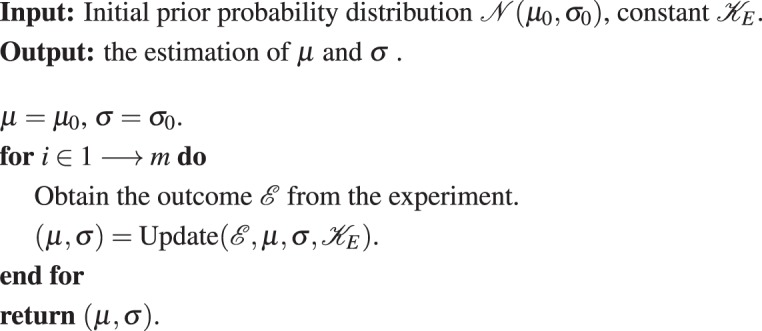
Bayesian phase estimation

**Algorithm 2:**
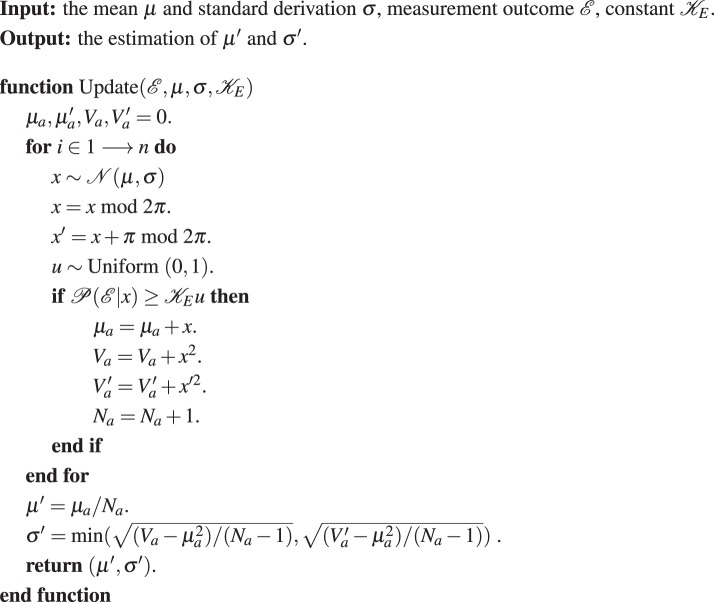
Updating function
